# A learning curve in using organ retractor for single-incision laparoscopic right colectomy

**DOI:** 10.1038/s41598-021-86168-4

**Published:** 2021-03-22

**Authors:** Toshio Shiraishi, Tetsuro Tominaga, Takashi Nonaka, Kiyoaki Hamada, Masato Araki, Yorihisa Sumida, Hiroaki Takeshita, Hidetoshi Fukuoka, Kazuo To, Kenji Tanaka, Terumitsu Sawai, Takeshi Nagayasu

**Affiliations:** 1 Departments of Surgical Oncology, Nagasaki University Graduate School of Biomedical Science, 1-7-1 Sakamoto, Nagasaki, 852-8501 Japan; 2 Department of Surgery, Sasebo City General Hospital, Sasebo, Japan; 3 Department of Surgery, National Hospital Organization Nagasaki Medical Center, Ōmura, Japan; 4 Department of Surgery, Isahaya General Hospital, Isahaya, Japan; 5 Department of Surgery, Ureshino Medical Center, Ureshino, Japan; 6 Department of Surgery, Saiseikai Nagasaki Hospital, Nagasaki, Japan

**Keywords:** Cancer, Gastrointestinal cancer, Metastasis, Tumour biomarkers

## Abstract

Single-incision laparoscopic surgery (SILS) has the potential to improve perioperative outcomes, including less postoperative pain, shorter operation time, less blood loss, and shorter hospital stay. However, SILS is technically difficult and needs a longer learning curve. Between April 2016 and September 2019, a total of 198 patients with clinical stage I/II right colon cancer underwent curative resection. In the case of the SILS approach, an organ retractor was usually used to overcome SILS-specific restrictions. The patients were divided into two groups by surgical approach: the SILS with organ retractor group (SILS-O, n = 33) and the conventional laparoscopic surgery group (LAC, n = 165). Clinical T status was significantly higher in the LAC group (*p* = 0.016). Operation time was shorter and blood loss was lower in the SILS-O group compared to the LAC group (117 vs. 197 min, *p* = 0.027; 10 vs. 25 mL, *p* = 0.024, respectively). In the SILS-O group, surgical outcomes including operation time, blood loss, number of retrieved lymph nodes, and postoperative complications were not significantly different between those performed by experts and by non-experts. Longer operation time (*p* = 0.041) was significantly associated with complications on univariate and multivariate analyses (odds ratio 2.514, 95%CI 1.047–6.035, *p* = 0.039). SILS-O was safe and feasible for right colon cancer. There is a potential to shorten the learning curve of SILS using an organ retractor.

## Introduction

Single-incision laparoscopic surgery (SILS) is the latest innovation in minimally invasive surgery^1^. In colorectal cancer patients, SILS reduces the risk of trocar-related complications and postoperative pain, requires shorter incisions, and improves cosmesis compared to conventional laparoscopic surgery^2–4^. Furthermore, a recent multicenter study showed that SILS shortens operation time, lowers blood loss, and shortens the hospital stay^5,6^.

On the other hand, SILS is often challenging and has some limitations because of the restricted movement of the surgical device, loss of triangulation, insufficient countertraction, and in-line viewing, which result in a longer learning curve^7,8^. In fact, current evidence for SILS in terms of right colectomy has been obtained from studies with the procedures performed by highly experienced surgeons^2,9–12^. Thus, the learning curve is the core issue to be resolved for SILS before it can become more commonly used worldwide.

To overcome these difficulties, we previously reported the effectiveness of SILS right colectomy using an organ retractor (B. Brown, Tokyo, Japan)^13^.

The aim of this multicenter study was to identify whether SILS right colectomy using an organ retractor was technically safe and has a shorter learning curve.

## Materials and methods

This multicenter, retrospective study was designed by the Nagasaki Colorectal Oncology Group (NCOG). Between April 2016 and September 2019, consecutive right colon cancer patients with clinical stages I and II were retrospectively reviewed if they underwent curative resection in the participating hospitals (Nagasaki University Hospital, Sasebo City General Hospital, Nagasaki Medical Center, Isahaya General Hospital, Ureshino Medical Center, and Saiseikai Nagasaki Hospital). Patients with incomplete laboratory data, synchronous colon cancer, open surgery, and emergency surgery were excluded. Finally, 198 patients were eligible for this analysis. The study was reviewed and approved by the Nagasaki university hospital clinical research ethics committee, Sasebo city general hospital clinical research ethics committee, Nagasaki Medical Center clinical research ethics committee, Isahaya General Hospital clinical research ethics committee, Ureshino Medical Center clinical research ethics committee, and Saiseikai Nagasaki Hospital clinical research ethics committee. The informed consent was obtained from all subjects. All methods were performed in accordance with the relevant guidelines and regulations.

**Figure 1:**
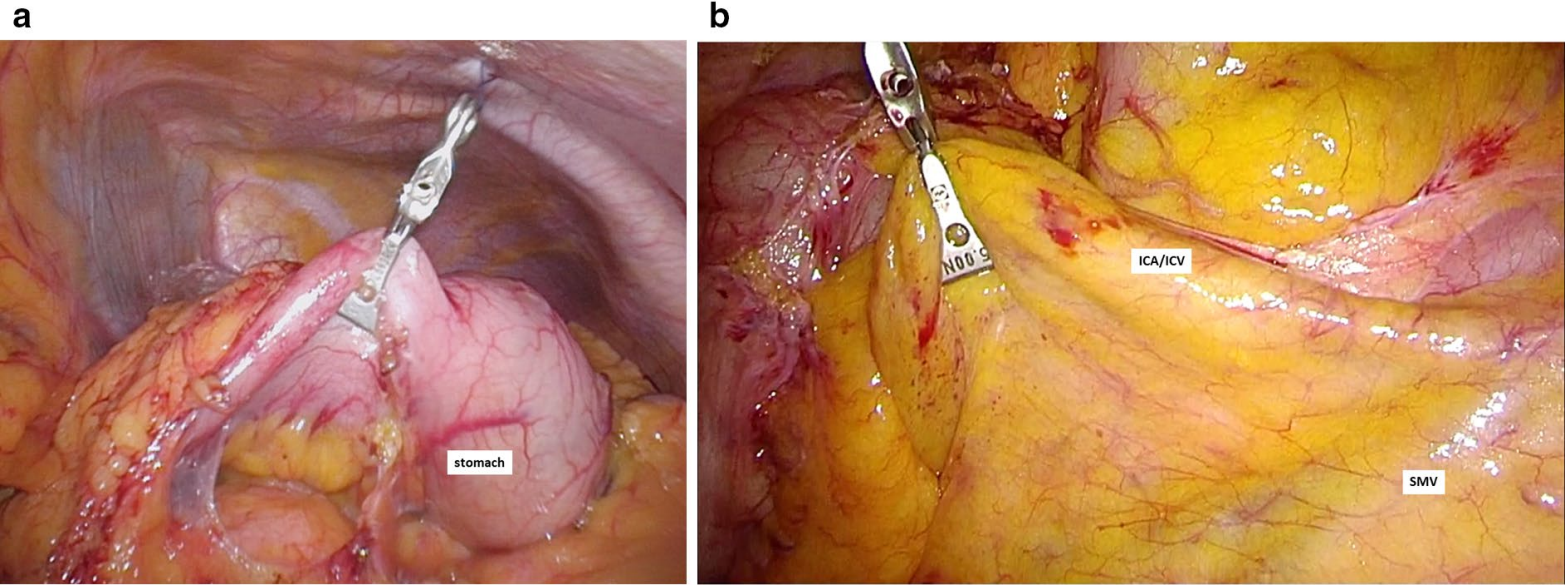
Operative procedure for single-incision laparoscopic right colectomy using an organ retractor. (**a**) To maintain the view of the hepatic flexure, the posterior wall of the stomach is grasped by the organ retractor, and it is trailed extracorporeally. (**b**) The pedicle of the ileocecal artery and vein is grasped by the organ retractor, and the vessels are dissected with the regional lymph nodes. ICA, ileocecal artery; ICV, ileocecal vein; SMV, superior mesenteric vein.

The SILS approach was performed as previously reported^13^. An organ retractor is a clothes peg-like device developed for grasping organs or tissue softly and gently. It can be de-installed using a remover that is used generically for intestinal grasp forceps. Since it is re-usable, it also has the advantage of low cost. A 3-cm incision was placed in the umbilicus. Then, EZ access (Hakko-medical, Tokyo, Japan) was inserted through the wound. Three ports, one for the scope and two for the handling forceps, were usually used. The procedure was usually started with the cranial approach for right colectomy, beginning with hepato-colic ligament resection. To maintain a good view of the hepatic flexure, the posterior wall of the stomach was grasped by the organ retractor (Fig. 1a). The organ retractor was trailed by Asflex (Crownjun, Chiba, Japan), which was inserted extracorporeally. After resection of the hepato-colic flexure, the pedicle of the ileocecal artery and vein was grasped by the organ retractor (Fig. 1b). Then, the regional lymph nodes and vessels were resected. To mobilize the intestine, the mesentery proper was grasped, and the insertion of the mesentery proper was cut. For each resection, the trailer line’s tension was adjusted to provide a stable surgical view. To remove the lesion from the body, the wound was dilated to 5 cm. The tumor was then resected by a suture instrument. A functional end-to-end anastomosis was made extracorporeally.

The patients were divided into two groups by surgical approach: the SILS with organ retractor group (SILS-O, n = 33) and the conventional laparoscopic surgery group (LAC, n = 165). The clinical features were compared between the groups. The following data were collected: sex, age at surgery, body mass index (BMI), American Society of Anesthesiologists (ASA)-performance status (PS), comorbidity, past history of abdominal surgery, tumor location, and clinical T status. Surgical and pathological data, including the type of reconstruction, number of retrieved lymph nodes, combined resection of adjacent organs, tumor size, operation time, estimated blood loss, percentage of operations performed by expert surgeons, histological type, pathological T status, pathological N status, presence or absence of lymphovascular invasion, postoperative complications, and postoperative hospital stay. Postoperative complications were defined as complications that occurred within 30 days of the primary surgery. Patients with Clavien-Dindo (CD) grade 2 or higher were included in the complication group.

In Japan, a training and certification system called the Japanese Endoscopic Surgical Skill Qualification System (JESSQS) has been established to objectively assess the skill of laparoscopic surgeons^14^. For JESSQS accreditation, applicant surgeons must submit their own unedited videos of high anterior resection or sigmoidectomy with lymph node dissection for colorectal cancer. The videos are assessed by two expert surgeons in a double-blinded fashion. They assess the display of the surgical field, autonomy of the operator, recognition of the anatomy, and cooperation of the surgical team. The qualification rate in the field of colorectal surgery is below 30% per year. The surgeons certified by the JESSQS not only possess advanced technical skills, but they are also capable of coaching trainees. In the present study, “expert surgeon” was defined as a surgeon who had acquired this certification in the colorectal field.

Statistical analysis was performed using Bell Curve for Excel software, version 2.02 (Social Survey Research Information Co., Ltd., Tokyo, Japan). The data are presented as median values with ranges. Differences in categorical variables were compared using Fisher’s exact test or the chi-squared test. Differences in continuous variables were analyzed with the Mann–Whitney U-test. Multivariate analysis using a Cox proportional hazards model was used to identify the independent risk factors for postoperative complications. All *p* values < 0.05 were considered significant.

## Results

Table 1 shows the clinicopathological characteristics of the 198 patients. The study population included 98 male and 100 female patients, with a median age of 74 (range 41–97) years. The median BMI was 22 (range 15–37) kg/m^2^, and 131 patients (66.2%) had poor PS (PS ≥ 2). Most patients had ascending colon cancer (n = 112, 56.6%), and 7 patients (16.6%) were diagnosed with clinical T4 preoperatively. Thirty-three patients (16.7%) underwent SILS-O. The median operation time and blood loss were 190 (range 70–385) min and 20 (0–560) mL, respectively. Thirty-four (17.1%) patients had postoperative complications.

**Table 1:** Patients’ characteristics.

	All patients (*n* = 198) (%)
**Sex**	
Male	98 (49.5)
Female	100 (50.5)
Age, y (range)	74 (41–97)
Body mass index, kg/m^2^	22 (15–37)
**ASA-performance status**	
1	67 (33.8)
2	115 (58.1)
3	16 (8.1)
Comorbidity, present	142 (71.7)
**Tumor location**	
Cecum	59 (29.8)
Ascending colon	112 (56.6)
Transverse colon	27 (13.6)
**Clinical T factor**	
1	97 (49.0)
2	45 (22.7)
3	49 (24.7)
4	7 (16.6)
SILS	33 (16.7)
Operation time, min (range)	190 (70–385)
Blood loss, mL (range)	20 (0–560)
Postoperative complications, CD ≥ 2	34 (17.1)

Data are presented as numbers of patients or medians (range).

*ASA* American Society of Anesthesiologists, *SILS* single-incision laparoscopic surgery.

Table 2 shows the clinical differences between the SILS-O group and the LAC group. Clinical T status was significantly higher in the LAC group (*p* = 0.016). Sex, age, BMI, ASA-PS, comorbidities, past history of abdominal surgery, and tumor location were similar between the two groups.

**Table 2:** Comparison of clinical characteristics between SILS and conventional LAC.

	SILS (*n* = 33) (%)	Conventional LAC (*n* = 165) (%)	*p* values
Sex			1.000
Male	16 (48.5)	82 (49.7)	
Female	17 (51.5)	83 (50.3)	
Age, y (range)	71 (51–84)	75 (41–97)	0.141
Body mass index, kg/m^2^	22 (18–27)	22 (15–37)	0.465
ASA-performance status			0.870
1	12 (36.4)	55 (33.3)	
2	19 (57.6)	96 (58.2)	
3	2 (6.0)	14 (8.5)	
Comorbidity			1.000
None	9 (27.3)	47 (28.5)	
Yes	24 (72.7)	118 (71.5)	
Past history of abdominal surgery			0.831
No	25 (75.8)	119 (72.1)	
Yes	8 (24.2)	46 (27.9)	
Tumor location			0.601
Cecum	8 (24.2)	51 (30.9)	
Ascending colon	19 (57.6)	93 (56.4)	
Transverse colon	6 (18.2)	21 (12.7)	
Clinical T status			0.016
1	24 (72.7)	73 (44.3)	
2	6 (18.2)	39 (23.6)	
3	3 (9.1)	46 (27.9)	
4	0 (0)	7 (4.2)	

Data are presented as numbers of patients or medians (range).

*ASA* American Society of Anesthesiologists, *SILS* single-incision laparoscopic surgery, *LAC* laparoscopic surgery.

Differences in categorical variables were compared using Fisher’s exact test or the chi-squared test, as appropriate. Differences in continuous variables were analyzed with the Mann–Whitney *U*-test.

Table 3 shows the surgical and pathological differences between the SILS-O group and the LAC group. Operation time was shorter and blood loss was lower in the SILS-O group compared to the LAC group (117 vs. 197 min, *p* = 0.027; 10 vs. 25 mL, *p* = 0.024, respectively). Regarding the remaining factors, including type of reconstruction, number of retrieved lymph nodes, conversion rate, tumor size, percentage of operations performed by expert surgeons, histological type, pathological T/N status, lymphovascular invasion, postoperative complications, and hospital stay, there were no significant differences between the two groups.

**Table 3:** Comparison of surgical and pathological characteristics between SILS and conventional LAC.

	SILS (*n* = 33) (%)	Conventional LAC (*n* = 165) (%)	*p* values
Reconstruction			0.745
Functional end-to-end anastomosis	31 (93.9)	148 (89.7)	
Hand sewn	2 (6.1)	17 (10.3)	
Retrieved lymph nodes, n (range)	15 (2–29)	16 (4–66)	0.113
Combined resection			0.520
No	32 (97.0)	162 (98.2)	
Yes	1 (3.0)	3 (1.8)	
Conversion to open surgery, yes	0 (0)	1 (0.6)	1.000
Tumor size, mm	20 (0–50)	28 (2–96)	0.060
Operation time, min (range)	177 (111–250)	197 (70–385)	0.027
Estimated blood loss, mL (range)	10 (0–100)	25 (0–560)	0.024
Performed by expert surgeon			0.058
No	19 (57.6)	123 (74.5)	
Yes	14 (42.4)	42 (25.5)	
Histological type			1.000
Well/moderate	32 (97.0)	158 (95.8)	
Poor/mucinous/signet	1 (3.0)	7 (4.2)	
Pathological T status			0.360
1–3	33 (100)	156 (94.5)	
4	0 (0)	9 (5.5)	
Pathological N status			0.264
Negative	31 (93.9)	142 (86.1)	
Positive	2 (6.1)	23 (13.9)	
Lymphovascular invasion			0.344
Negative	19 (57.6)	79 (47.9)	
Positive	14 (42.4)	86 (52.1)	
Postoperative complications (CD ≥ 2)			0.460
No	26 (78.8)	138 (83.6)	
Yes	7 (21.2)	27 (16.4)	
Postoperative hospital stay, days (range)	10 (6–54)	13 (7–54)	0.499

Differences in categorical variables were compared using Fisher’s exact test or the chi-squared test, as appropriate. Differences in continuous variables were analyzed with the Mann–Whitney *U*-test.

*CD* Clavien-Dindo grade, *SILS* single-incision laparoscopic surgery, *LAC* laparoscopic surgery.

Table 4 shows the clinical and surgical characteristics of the patients who underwent SILS-O right colectomy by an expert or by a non-expert surgeon. Patients’ characteristics such as sex, age, BMI, and clinical T status were similar between the groups. Surgical outcomes including operation time, blood loss, number of retrieved lymph nodes, postoperative complications, and hospital stay were not significantly different between the expert and non-expert groups. Table 5 shows the clinical and surgical characteristics of the patients who underwent LAC right colectomy by an expert or by a non-expert surgeon. There were more patients with locally advanced tumor (T4) in the expert group (11.9% vs. 1.6%, *p* = 0.009). Other factors were not significantly different between the groups.

**Table 4:** Comparison of clinical characteristics in SILS performed by an expert or a non-expert.

	Expert (*n* = 14) (%)	Non-expert (*n* = 19) (%)	*p* values
Sex			0.166
Male	9 (64.3)	7 (36.8)	
Female	5 (35.7)	12 (63.2)	
Age, y (range)	71 (57–84)	72 (51–81)	0.720
Body mass index, kg/m^2^	22 (18–27)	22 (18–26)	0.544
Clinical T status			0.883
1	10 (71.4)	14 (73.7)	
2	3 (21.4)	3 (15.8)	
3	1 (7.2)	2 (10.5)	
Operation time, min (range)	175 (111–244)	179 (127–250)	0.214
Estimated blood loss, mL (range)	13 (0–100)	10 (0–76)	0.568
Retrieved lymph nodes (range)	16 (7–29)	15 (2–25)	0.635
Postoperative complications (CD ≥ 2)			0.106
No	9 (64.3)	17 (89.5)	
Yes	5 (35.7)	2 (10.5)	
Hospital stay (range)	9 (6–41)	10 (7–54)	0.801

Data are presented as numbers of patients or medians (range).

Differences in categorical variables were compared using Fisher’s exact test or the chi-squared test, as appropriate. Differences in continuous variables were analyzed with the Mann–Whitney *U*-test.

*SILS* single-incision laparoscopic surgery.

**Table 5:** Comparison of clinical characteristics in LAC performed by an expert or a non-expert.

	Expert (*n* = 42) (%)	Non-expert (*n* = 123) (%)	*p* values
Sex			1.000
Male	21 (50.0)	61 (49.6)	
Female	21 (50.0)	62 (50.4)	
Age, y (range)	77 (41–97)	74 (41–94)	0.119
Body mass index, kg/m^2^	23 (17–34)	23 (15–37)	0.503
Clinical T status			0.009
1	22 (52.4)	51 (41.5)	
2	6 (14.3)	33 (26.8)	
3	9 (21.4)	37 (30.1)	
4	5 (11.9)	2 (1.6)	
Operation time, min (range)	215 (121–385)	192 (70–371)	0.755
Estimated blood loss, mL (range)	25 (0–560)	23 (0–476)	0.755
Retrieved lymph nodes, n (range)	18 (7–29)	16 (2–25)	0.483
Postoperative complications (CD ≥ 2)			1.000
No	35 (83.3)	103 (83.7)	
Yes	7 (16.7)	20 (16.3)	
Hospital stay (range)	18 (7–38)	16 (7–54)	0.483

Data are presented as numbers of patients or medians (range).

Differences in categorical variables were compared using Fisher’s exact test or the chi-squared test, as appropriate. Differences in continuous variables were analyzed with the Mann–Whitney *U*-test.

*LAC* laparoscopic surgery.

The overall postoperative complication rate (CD ≥ 2) was 17.1% (n = 34) (Table 6). Of these 34 patients with morbidities, 21 (61.9%) had grade II complications, including paralytic ileus (n = 10), delirium (n = 3), leakage (n = 2), surgical site infection (n = 2), pseudomembranous colitis (n = 1), urinary tract infection (n = 1), chylous ascites (n = 1), and thrombosis (n = 1). Thirteen patients had grade III or higher complications, including leakage (n = 5), bowel obstruction (n = 4), anastomotic bleeding (n = 2), and surgical site infection (n = 2). There were no significant differences between the SILS-O and LAC groups.

**Table 6:** Details of the postoperative complications of the 34 patients.

		SILS	Conventional LAC	*p*-value
**Number**	34	7	27	0.460
CD Grade 2	21 (61.9%)	4 (57.1)	17 (63.0)	0.758
Paralytic ileus	10	2	8	
Delirium	3	1	2	
Leakage	2	1	1	
Surgical site infection	2	0	2	
Pseudomembranous colitis	1	0	1	
Urinary tract infection	1	0	1	
Chylous ascites	1	0	1	
Thrombosis	1	0	1	
CD Grade 3	11 (32.3%)	3 (42.9)	8 (29.6)	0.397
Bowel obstruction	3	1	2	
Leakage	3	0	3	
Anastomotic bleeding	3	2	1	
Surgical site infection	2	0	2	
CD Grade 4	1 (2.9%)	0 (0)	1 (3.7)	1.000
Leakage	1	0	1	
CD Grade 5	1 (2.9%)	0 (0)	1 (3.7)	1.000
Leakage	1	0	1	

*CD* Clavien-Dindo grade.

Table 7 shows the results of univariate and multivariate analyses of risk factors for postoperative complications. Longer operation time (*p* = 0.041) was significantly associated with complications on univariate analysis. Multivariate analysis also showed that operation time (odds ratio 2.514, 95%CI 1.047–6.035, *p* = 0.039) was an independent predictor.

**Table 7:** Clinical factors predicting postoperative complications of colorectal cancer patients with synchronous distant metastases.

	Univariate analysis	Multivariate analysis		
	*p* value	Odds ratio	95% CI	*p* value
Sex	0.119			0.305
Female		1		
Male		1.512	0.6853.340	
Age, y	0.669			
< 80				
≥ 80				
ASA-performance status	0.129			
1–2				
3				
BMI, kg/m^2^	0.191			
< 25				
≥ 25				
Clinical T status	0.837			
1–3				
4				
Comorbidities	0.136			
No				
Yes				
Past history of abdominal surgery	0.758			
No				
Yes				
Operative procedure	0.501			
Laparoscopic				
SILS				
Reconstruction	0.179			
Hand sewn				
Functional end-to-end anastomosis				
Operation time, min	0.041			0.039
< 180		1		
≥ 180		2.514	1.047–6.035	
Estimated blood loss, mL	0.058			0.234
< 50		1		
≥ 50		0.610	0.270–1.377	
Performed by expert surgeon	0.320			
No				
Yes				

*HR* hazard ratio, *CI* confidence interval, *ASA* American Society of Anesthesiologists.

A Cox proportional hazards model was used to identify the independent risk factors for postoperative complications.

## Discussion

In the present study, SILS-O had a shorter operation time and less blood loss with sufficient lymph node dissection compared to conventional LAC. The postoperative complication rate was not increased by this procedure. Furthermore, non-expert surgeons could perform SILS safely by using an organ retractor, which suggests a better learning curve with this approach. To the best of our knowledge, this is the first report to evaluate short-term outcomes and the effect on the learning curve of the SILS-O approach.

Previous randomized, controlled trials have evaluated the short-term outcomes of SILS compared to conventional LAC^15,16^. SILS has potential advantages, including shorter operation time, less blood loss, less postoperative pain, and shorter hospital stay. In patients with right-side colon cancer, Ishii and colleagues examined 65 patients with right-side colon cancer and evaluated the short-term and mid-term outcomes of the SILS approach^17^. The median operation time and blood loss were 216 min and 10 mL, respectively. Liu et al. reviewed 1,356 patients who participated in 9 studies and performed a meta-analysis to evaluate the effects of SILS and conventional LAC in right-side colon cancer^18^. Similar to the previous study, operation time was shorter and blood loss was less in the SILS group compared to conventional LAC.

On the other hand, SILS is a difficult technique that requires advanced laparoscopic skill, instrumentation, and maintenance of the operative field^19^. There is a significant learning curve compared to conventional LAC^20^. In fact, recent evidence for SILS in right colectomy has been obtained from studies with the procedures performed by experienced surgeons in high-volume centers^2,9,10^. Difficulty with the learning curve is a serious problem for SILS to become more commonly used worldwide. The six participating hospitals in the present multicenter study are all low-volume centers, with < 200 CRC surgeries performed annually. However, the present results showed that SILS using an organ retractor resulted in shorter operation time (117 min) and less blood loss (10 mL) compared to previous reports of SILS without using an organ retractor (168–217 min and 41–134 mL, respectively)^4,18,21,22^. One possible explanation is that the surgical indication in the present study was limited to clinical stage I/II patients and did not include large tumors. Another possible explanation for this result is that the organ retractor could maintain a good operative field in any situation, including vessel dissection and bowel mobilization, which enabled a smooth process throughout the operation.

In the present study, “expert surgeon” was defined as a surgeon with JESSQS certification. In general, in conventional laparoscopic surgery, an expert could help a non-expert operator as a first assistant during the operation. On the other hand, in the SILS cohort, the non-expert operator should have completed the surgery without receiving an expert’s help. Of the 33 patients who underwent SILS-O, about 60% of cases were performed by “non-expert surgeons”. Even in this situation, perioperative outcomes including operation time, blood loss, postoperative complications, and hospital stay were similar between the “expert” and “non-expert” groups (Table 4). Furthermore, the number of harvested lymph nodes is important in cancer surgery, and there was no significant difference between the groups. We hypothesized that these results show that non-expert surgeons could perform SILS-O safely, and an organ retractor could be a useful instrument to shorten the learning curve.

The unexpected open conversion rate of SILS has been reported to range from 1.4 to 9.5%^4,18,21,22^. Furthermore, the insertion rate of additional ports was 2% to 28% because of dense adhesions, limited working space, and to maintain surgical quality^16,17,23,24^. Conversion of laparoscopic to open surgery could increase postoperative complications and result in a longer hospital stay^25^.

To overcome intraoperative restrictions, the application of SILS plus one-port laparoscopic surgery (SILS + 1) has recently attracted attention^7,26–31^. Indeed, the conversion rate of the SILS + 1 approach (1.1–4.9%) was reported to be lower than that of SILS^29,32^. However, insertion of an additional port could risk injury of the intestine, bleeding, herniation, and postoperative pain. In the present study, no patients required open conversion or an additional port in the SILS-O group. An organ retractor has the potential for not only performing safe SILS surgery, but also reducing port-related complications.

There were some limitations in the present study. First, it was a retrospective study, and the patient cohort was heterogenous, with several selection biases. A propensity score-matched analysis with a much larger cohort or a prospective, randomized, controlled trial would be needed to confirm the present results. Second, in colon cancer, complete mesocolic excision (CME) is a crucial concept to secure good oncological outcomes^33^. The rationale underlying CME is complete resection of the colon and intact mesocolon. An organ retractor is an instrument that grasps the tissue safely and gently. In fact, in no cases did the organ retractor injure the surrounding tissues in the present study. However, no reports have evaluated the long-term oncological outcomes of surgery using an organ retractor. Further evaluation is needed to resolve these issues. Third, the operation time, blood loss, and the rate of postoperative complications were not significantly different between LAC performed by an expert and that by a non-expert (Table 5). One possible explanation is that there were more patients with locally advanced tumor (T4) in the expert group (11.9% vs. 1.6%, *p* = 0.009). Another explanation is that, in the conventional LAC cohort, 55% (67/123) of the operations were performed by a non-expert surgeon supervised by an experienced surgeon as a first assistant.

Even taking these limitations into account, SILS using an organ retractor is one of the options for performing surgery safely for patients with right-side colon cancer.
